# Metabolomic and transcriptomic analyses reveal the mechanisms underlying *Smilax glabra* seed dormancy release

**DOI:** 10.3389/fgene.2026.1814199

**Published:** 2026-05-01

**Authors:** Haidi Wang, Danhong Zhong, Feng Wang, Siqi Zheng, Xiaoming Sun, Yuxin Pang, Yuan Yuan, Xiaomin Tang

**Affiliations:** 1 School of Traditional Chinese Medicine, Guangdong Pharmaceutical University, Guangzhou, China; 2 Deqing Forest Farm of Guangdong Province, Zhaoqing, Guangdong, China; 3 Key Laboratory of Production and Development of Cantonese Medicinal Materials, State Administration of Traditional Chinese Medicine, Guangzhou, China; 4 Comprehensive Experimental Station of Guangzhou, Chinese Material Medica, China Agriculture Research System (CARS-21-16), Guangzhou, China; 5 College of Pharmacy, Guizhou University of Traditional Chinese Medicine, Guiyang, China

**Keywords:** *Smilax glabra*, seed dormancy, transcriptome and metabolome, hormone, starch and sucrose metabolism

## Abstract

*Smilax glabra*, a perennial vine with high pharmaceutical value, exhibits seed dormancy under natural conditions that severely restricts artificial propagation and industrial-scale cultivation. This study aims to systematically investigate the physiological and molecular mechanisms underlying temperature-stratification-induced (25 °C/4 °C) dormancy release in *S. glabra* seeds, providing a scientific basis for optimizing seed propagation protocols. In this study, ultraviolet spectrophotometry (UV) was employed to monitor the dynamic variations in soluble sugars, starch, soluble protein content, and enzymatic activities (including phosphoglucose isomerase (PGI), malate dehydrogenase (MDH), and glucose-6-phosphate dehydrogenase (G6PDH)) during temperature stratification (25 °C/4 °C) of *S. glabra* seeds. Additionally, ultra-performance liquid chromatography-tandem mass spectrometry (UPLC-MS/MS) and RNA-seq were integrated to systematically characterize the phytohormone metabolic profiles and molecular biological mechanisms underlying seed stratification. The results demonstrated that alternating temperatures of 25 °C/4 °C effectively broke seed dormancy. During the dormancy release process, seeds consumed soluble proteins and starch, while starch-degrading genes (*AMY* and *BAM*) were significantly upregulated to drive starch conversion into soluble sugars. Meanwhile, PGI activity exhibited a marked decrease, while MDH and G6PDH activities showed overall upward trends. At the hormone metabolism level, downregulation of *NCED* and upregulation of *CYP707A* synergistically reduced ABA levels, while upregulation of *GA20ox* and *KAO* promoted GA biosynthesis; downregulation of *DELLA* protein-encoding genes relieved GA signal suppression, collectively forming the “ABA decline-GA rise” hormonal balance shift that drives seed germination. Simultaneously, JA biosynthesis genes *LOX2* and *AOS* were upregulated, leading to OPDA accumulation, but OPDA was not converted into JA. Furthermore, the upregulation of the negative regulator *JAZ* blocked JA signal transduction, thereby relieving JA-mediated inhibition of germination. This study reveals the molecular mechanisms underlying 25 °C/4 °C temperature-stratification-mediated dormancy release in *S. glabra* seeds through integrated physiological, metabolic, and transcriptional analyses.

## 1 Introduction

*Smilax glabra* is a medicinal plant belonging to the Liliaceae family. The rhizome is efficacious in detoxification, dampness resolution, and joint function promotion, and is clinically used to treat damp-heat syndrome, leukorrhea, abscesses, scrofula, scabies, bone and tendon pain, as well as limb contractures caused by syphilis or mercury poisoning (Pharmacopoeia of the People’s Republic of China, 2025). Phytochemical analyses have identified that *S. glabra* contains substantial quantities of astilbin, resveratrol, isoengeletin, and neoastilbin (ZOU et al., 2024). Modern pharmacological studies have demonstrated that these compounds exhibit antineoplastic effects (FU et al., 2024), ameliorate cardiac hypertrophy, and attenuate myocardial inflammation (Jing-lei and HOU, 2021). In recent years, with the expanding applications of *S. glabra‘s* medicinal functions, market demand has grown steadily, leading to depletion of wild resources and supply shortages (DONG et al., 2014). Consequently, artificial cultivation has become an essential measure to meet market demand, with seedling supply being the key prerequisite for successful cultivation. The species principally relies on seed propagation. The seed structure, characterized by a thin seed coat that tightly adheres to the endosperm surrounding the embryo, which is very small, is similar to that of *Acanthopanax senticosus* (Yanjun et al., 2024). Studies demonstrate that the developmental status of the embryo and the presence of endogenous germination inhibitors are the primary factors responsible for seed dormancy in *A. senticosus* (Yanjun et al., 2024). The role of seed dormancy factors in *S. glabra* remains unclear. Nevertheless, the seeds display pronounced dormancy traits, including extended germination periods and low germination rates, which substantially hinder nursery production. Thus, the establishment of effective dormancy-breaking protocols is essential. Stratification, as a commonly employed method in agricultural and horticultural fields for breaking seed dormancy, is essential for enhancing the efficiency of seedling propagation and the quality of seedlings (Song et al., 2021). Low-temperature stratification can overcome the inhibitory effect of the endosperm and promote embryo growth in *Epimedium koreanum* (Zhang et al., 2024). For *Sinopodophyllum hexandrum* seeds, low-temperature stratification not only promotes embryo morphological development but also regulates nutrient transformation and accumulation (e.g., starch, soluble proteins, and sugars) while affecting related metabolic enzyme activity (Ning et al., 2024). The seeds of *A. senticosus* display morphophysiological dormancy (Yanjun et al., 2024). while the constant temperature of 20 °C only promoted embryo elongation, but not germination (Yanjun et al., 2024). There was no increase in embryo rate at 4 °C, and the embryo rate increased, and the seeds germinated only when the temperature was maintained at 20 °C for 0–110 days and 4 °C for 110–140 days (Yanjun et al., 2024). Given the structural similarity between *A. senticosus* and *S. glabra* seeds, a sequential warm-to-cold stratification offers a promising approach to addressing dormancy in *S. glabra* seeds.

Seed dormancy release and germination constitute a complex physiological process involving dynamic changes in endogenous substance metabolism and energy supply. During this process, starch, lipids, and proteins are respectively degraded into soluble, fatty acids, and amino acids, establishing the material basis for seed germination (DONG et al., 2014; Zhang et al., 2022). Enzymes and related genes linked to starch-sucrose conversion have been extensively researched in several species (MacNeill et al., 2017). Emerging evidence indicates that sucrose synthase (*SUS*/*SS*) and α-amylase (*AMY*) hydrolyze sucrose and starch into glucose during Callery Pear seed dormancy release, thereby directly promoting dormancy breakage (Zhang et al., 2022). Seed germination relies on energy provided by three major respiratory metabolic pathways: the glycolytic pathway (EMP), the tricarboxylic acid (TCA), and the pentose phosphate pathway (PPP). The key enzymes of these pathways are phosphoglucose isomerase (PGI), malate dehydrogenase (MDH), and glucose-6-phosphate dehydrogenase (G-6-PDH), respectively. The activity levels of these enzymes directly indicate the operational status of their corresponding respiratory pathways (Zhu et al., 2023; Liu et al., 2025). Roberts et al. (Roberts, 1969) proposed that the transition of respiratory metabolism from EMP to PPP is concurrent with the conversion of NAD to NADPH, which thereby facilitates seed dormancy release. This indicates that the regulation of respiratory metabolic pathways likely plays a pivotal role in breaking seed dormancy.

Beyond material and energy metabolism, phytohormones serve as pivotal signaling molecules governing seed dormancy induction, maintenance, and release (Chang et al., 2018). Among these phytohormones, abscisic acid (ABA) and gibberellins (GA) serve as the predominant hormonal regulators in controlling seed dormancy and germination, whereas auxin (AUX), cytokinine (CTK), brassinosteroid (BR), ethylene (ET), jasmonic acid (JA), and salicylic acid (SA) generally exert their regulatory effects by interacting with ABA or GA signaling pathways (Zhao et al., 2024a). Specifically, ABA induces and maintains seed dormancy, whereas GA counteracts ABA to promote dormancy release and germination (Longo et al., 2020; Sano and Marion-Poll, 2021; Ali et al., 2022). The accumulation of abscisic acid (ABA) is primarily regulated by two key enzymes: 9-cis-epoxycarotenoid dioxygenase (*NCED*) and cytochrome P450 monooxygenase (*CYP707A*). The downregulation of *NCED* in ABA biosynthesis and the upregulation of *CYP707A* in ABA catabolism contribute to seed dormancy release (Sano and Marion-Poll, 2021). GA promotes seed germination by triggering the degradation of *DELLA* proteins, which function as suppressors in the signaling pathway, thereby relieving *DELLA*-mediated inhibition (Gao et al., 2025).

Despite substantial advances in elucidating seed dormancy regulation in diverse plant species, the exact physiological and molecular mechanisms governing dormancy release in *S. glabra* seeds remain to be elucidated. Co-analysis of transcriptomics and metabolomics can connect phenotype alterations to molecular mechanisms, elucidating the intricate biological processes and regulatory networks engaged in seed dormancy release (Fu et al., 2021; Chen et al., 2023). Prior research has confirmed that seed dormancy and germination are co-regulated by hormone signaling transduction, starch-sucrose metabolism, and respiratory metabolism (including EMP, TCA, and PPP) (Pan et al., 2023). Building on this foundation, this study firstly employs physiological-biochemical assays, transcriptomics, and hormonal metabolomics analyses to systematically examine how stratification affects key metabolic pathways, differentially expressed genes, and differential metabolites in *S. glabra* seeds, aiming to reveal the molecular mechanisms by which stratification breaks seed dormancy. Furthermore, this study contributes scientific evidence for optimizing seed propagation techniques and advancing sustainable *S. glabra* cultivation, while providing valuable insights for seed storage and seedling production in other plant species.

## 2 Materials and methods

### 2.1 Plant material collection and seed treatment

The *S. glabra* seeds used in this research were collected in April 2024 from cultivated plants in Hezhou City, Guangxi Province, China (elevation 1000–1700 m; 111°34′–112°04′E, 23°39′–25°09′N). The seeds were authenticated by Associate Professor Xiaomin Tang from the School of Traditional Chinese Medicine, Guangdong Pharmaceutical University. Mature seeds were surface-sterilized by immersion in 0.1% carbendazim solution for 15 min, followed by three sequential rinses with sterile water, and air-dried for subsequent use. Vermiculite was autoclaved at 121 °C (105 kPa) for 30 min. The seeds were mixed with moistened vermiculite (humidity standard: retains form when compact but disperses upon release) at a 1:3 (v/v) ratio in germination boxes. Primary stratification was performed at 25 °C for 180 days, followed by secondary stratification at 4 °C. During stratification, seeds were periodically turned to ensure aeration and sprayed with purified water to maintain 70% humidity. Sampling was conducted at 0, 60, 120, 180, 240, 270, 300, and 330 days (D0, D60, D120, D180, D240, D270, D300, and D330) of stratification. For each time point, three biological replicates (20.00 g per replicate) were collected, quick-frozen in liquid nitrogen, and kept at −80 °C for subsequent physiological, transcriptomic, and metabolomic studies.

### 2.2 Seed viability assay

Seed viability was evaluated using the 2,3,5-triphenyltetrazolium chloride (TTC) staining assay. Fifty seeds were randomly selected, bisected longitudinally to expose the embryo, and transferred to amber glass bottles. Seed halves were subsequently incubated in 0.5% TTC solution at 25 °C in darkness, with the volume sufficient to fully immerse the samples. After 6 h of staining, samples were removed and rinsed thrice with distilled water before embryo coloration assessment. Red-stained embryos were scored as viable, while unstained embryos were classified as non-viable. All treatments were performed in triplicate.

### 2.3 Determination of germination rate and physiological indexes

For each stratification period (D0, D60, D120, D180, D240, D270, D300, and D330), 300 seeds of *S. glabra* were randomly selected for germination experiments. Germination was determined as the protrusion of the radicle from the seed coat. The seeds were placed in glass culture dishes (d = 9 cm) lined with two layers of filter paper. Each replicate contained 100 seeds, with three replicates performed. The Petri dishes were incubated in a BSPX-400 GB illuminated incubator (Shanghai, China) at 25 °C, with a 16-h light/8-h dark cycle and 70% relative humidity. Sterile water was sprayed daily to maintain humidity levels. Germinated seeds (defined by radicle emergence) were recorded daily. The rate of germination was determined by the formula:

$$Germination\;Rate\;\left(\%\right)=\left(Number\;of\;Germinated\;Seeds\;/\;Total\;Number\;of\;Test\;Seeds\right)\times 100\%$$

The soluble sugar content was measured using the anthrone colorimetric method, while the soluble protein content was measured using the Coomassie Brilliant Blue method. Starch content, MDH activity, and G6PDH activity were assayed with kits from Beijing Solarbio Science & Technology Co., Ltd. PGI activity was analyzed using kits from Shenzhen Ruiqin Biological Technology Co., Ltd. All measurements were performed in triplicate.

### 2.4 Phytohormone metabolite profiling and data analysis

The 41 phytohormones of *S. glabra* seeds in three specific stratification stages (D0, D270, and D300) were measured quantitatively using liquid chromatography-tandem mass spectrometry (LC-MS/MS). The integrated system for chromatographic separation and mass spectrometric detection consisted of an ultra-performance liquid chromatography (UPLC) system (ExionLC™ AD; Shanghai, China) coupled with a tandem mass spectrometer (QTRAP® 6500+; Shanghai, China). Chromatographic conditions: Separation was performed on a Waters BEH C18 column (2.1 × 100 mm, 1.7 μm) maintained at 30 °C. The mobile phase included (A) 0.1% formic acid in water and (B) 0.1% formic acid in acetonitrile. The injection volume was 10 μL with a flow rate of 0.35 mL/min. The gradient elution program was as follows: 0.0–3.0 min, 30%–60% B; 3.0–10.0 min, 60%–100% B; 10.0–12.0 min, 100% B (isocratic). Mass spectrometric conditions: Data were collected in both positive and negative ion modes. The ion source parameters were set as follows: curtain gas (CUR) at 35 psi, collision gas (CAD) at medium level, ion spray voltage (IS) at +5500 V (positive mode)/-4500 V (negative mode), and temperature (TEM) at 550 °C. The ion source gases were set at 50 psi for both GS1 and GS2. Scheduled multiple reaction monitoring (MRM) was employed with a detection window of 120 s.

KEGG enrichment analysis was conducted on the identified metabolites. Principal component analysis (PCA) and hierarchical cluster analysis (HCA) were performed to assess sample variations based on the processed data. To identify differentially regulated metabolites between groups, the absolute log2-fold change (log2FC) values of metabolite expression levels were calculated. Differentially expressed hormone metabolites (DHMs) were screened using a threshold of |log2FC| > 1 (FC > 2). These metabolites were subsequently subjected to one-way ANOVA, and those with p < 0.05 were considered significant DHMs.

### 2.5 Transcriptome sequencing and analysis

Transcriptome sequencing was performed by Shanghai Majorbio Bio-pharmTechnology Co., Ltd. Nine samples were used to extract total RNA, which was then evaluated for concentration and purity using a Nanodrop 2000, integrity assessed by agarose gel electrophoresis, and RNA quality number (RQN) assessed with an Agilent 5300. Library construction required ≥1 μg RNA (concentration ≥30 ng/μL), an RQN >6.5, and OD260/280 ratios of 1.8–2.2. High-quality RNA was used for cDNA library preparation, followed by sequencing on an Illumina high-throughput platform.

Sequencing image data was transformed into nucleotide sequences by CASAVA base calling and stored as raw reads in FASTQ format. Transcript sequences were obtained by filtering raw data with fastp (v0.23.4) and assembling them with Trinity (v2.8.5). *De novo* assemblies were filtered and optimized using TransRate (v1.0.3), and redundancy was removed with CD-HIT (v4.5.7) to generate non-redundant sequences. Functional annotation was achieved by aligning transcripts against six databases (NR, Swiss-Prot, Pfam, CO, GO, and KEGG) using DIAMOND (v2.1.9) and HMMER (v3.2.1), followed by statistical analysis of annotations. Hierarchical clustering of samples was carried out using the R package pheatmap. Differential expression analysis was conducted utilizing DESeq2 (v1.42), with significant levels set at |log2FC| ≥ 1 and Padj <0.05. Gene Ontology (GO) and KEGG pathway enrichment analyses were subsequently conducted for DEGs from comparative groups.

### 2.6 Quantitative real-time PCR (qRT-PCR)

The identical RNA samples used for RNA-seq were also used for qRT-PCR validation. 18S rRNA served as the reference gene (internal control) in the study of twelve DEGs. Reactions were conducted utilizing SYBR Green QPCR Mix (Kejin Biotechnology, Chengdu, China) on a real-time PCR platform. The relative gene expression levels were calculated by the 2^−ΔΔCT^ method. Each sample contained three biological duplicates. Details of all primers used are shown in Supplementary Table S1.

### 2.7 Integrated analysis of metabolome and transcriptome

DEGs and DAMs were analyzed for their biological pathways using KEGG enrichment. Co-expression networks were constructed utilizing the WGCNA package in R, and eigengene values were computed for each module to examine their relationships with specific hormone-related metabolites. For the correlation analysis between metabolites and genes, the Pearson correlation coefficient was determined, and the results were visualized using R software (version 2.10).

### 2.8 Statistical analysis

All experiments were conducted with three independent biological replicates, and data analysis was performed using SPSS version 27.0. For multiple comparisons, a one-way ANOVA followed by Tukey’s HSD *post hoc* test was employed. A p-value of less than 0.05 was considered statistically significant among different stratification stages.

## 3 Results

### 3.1 Physiological changes during dormancy release in *Smilax glabra* seeds

**FIGURE 1:**
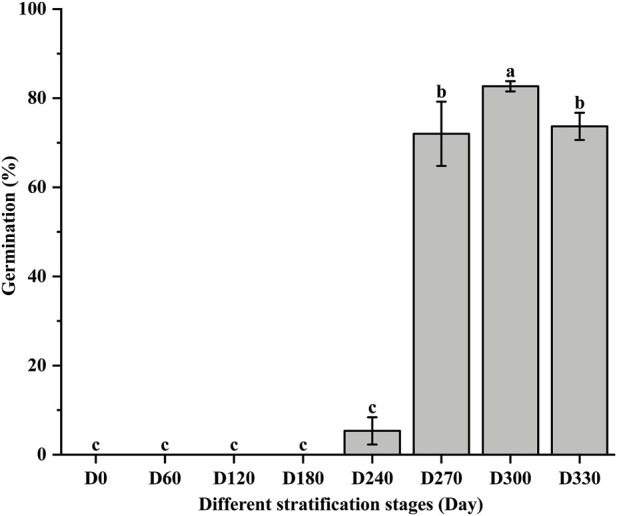
Germination rate of *Smilax glabra* seeds at various stratification stages.

**FIGURE 2:**
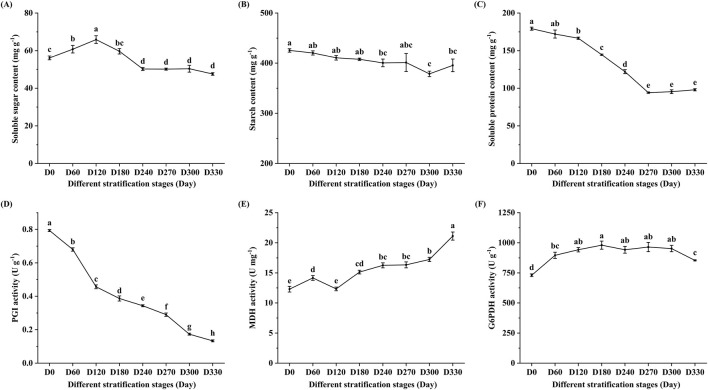
Changes in seed physiological characteristics of *Smilax glabra* during stratification. **(A)** Soluble sugar content. **(B)** Starch content. **(C)** Soluble protein content. **(D)** Phosphoglucose isomerase (PGI) activity. **(E)** Malate dehydrogenase (MDH) activity. **(F)** Glucose-6-phosphate dehydrogenase (G6PDH) activity. Values represent mean ± standard deviation (n = 3) from three independent biological replicates. Means with different letters differ significantly (p < 0.05).

Assessment revealed that 90.0% of seeds were viable. Germination of *S. glabra* seeds was initiated at D240, achieving a germination rate of 5.3%. The germination rate rose significantly to 72.0% at D270 and reached its maximum of 82.7% at D300 (Figure 1). Figures 2A–C illustrates the temporal variations in nutrient content under alternating-temperature stratification. Soluble sugar content displayed a temporary accumulation followed by a subsequent reduction, attaining its maximum of 65.88 mg·g^-1^ at D120, which represented a 17.37% increase compared to the pre-stratification level (56.13 mg·g^-1^). At D240, soluble sugar content dropped sharply to 50.22 mg·g^-1^, followed by a gradual decline. Starch content showed an overall downward trend, remaining relatively stable from D0 to D270 before declining significantly (p < 0.05) at D300. A partial recovery was observed at D330, though the content remained below the D0 level. Soluble protein content decreased progressively, with a rapid reduction from D0 to D270, after which the levels stabilized. Compared to the pre-stratification content (179.22 mg·g^-1^), protein content at D270 (94.18 mg·g^-1^) decreased by 47.45%. These results indicate that alternating-temperature stratification (25 °C/4 °C) effectively breaks dormancy in *S. glabra* Roxb. seeds. During dormancy release, soluble sugars displayed transient accumulation followed by depletion, while starch and soluble protein were progressively utilized.

Figures 2D–F illustrates the changes in the enzyme activities of key respiratory pathway enzymes under alternating-temperature stratification of *S. glabra* seeds. PGI activity exhibited a continuous decline. The highest PGI activity (0.79 U·g^-1^) was observed in non-stratified seeds, while it significantly decreased to its lowest value (0.13 U·g^-1^) on D330 during stratification. G6PDH activity displayed an initial increase followed by a decrease, peaking at 980.49 U·g^-1^ on D180, representing a 34.21% increase compared to pre-stratification levels (730.54 U·g^-1^). MDH activity exhibited an overall upward trend, reaching its maximum (21.12 U·g^-1^) on D330, representing a 71.85% increase compared to the initial level (12.29 U·g^-1^). These results indicate that PGI enzyme activity significantly decreased, while MDH and G6PDH enzyme activities increased during dormancy release in *S. glabra* seeds.

### 3.2 Metabolomic analysis during seed dormancy release in *Smilax glabra*

**FIGURE 3:**
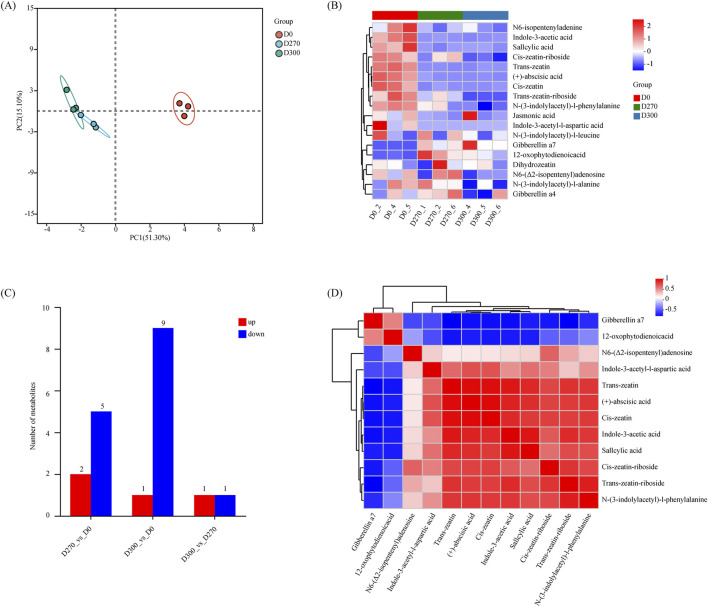
Metabolite profile analysis of *Smilax glabra* seeds at three stratification stages (D0, D270, D300). Principal Component Analysis (PCA) of metabolites **(A)**. Comprehensive cluster heatmap analysis of total metabolites **(B)**. Bar graph of total differentially expressed metabolites (DEMs) expression profiles **(C)**. Correlation heatmap of DEMs **(D)**.

To investigate the roles of endogenous hormones in *S. glabra* seeds, 33 phytohormones were measured, among which 18 metabolites were detected, while the levels of other hormones were below the detection limit (Table 1). PCA analysis showed distinct separation of samples from the three stages (D0, D270, and D300) along PC1 and PC2, with replicates from the same stage clustering together (Figure 3A). Heatmap-based analysis of the identified phytohormone levels revealed significant fluctuations in their expression levels (Figure 3B). Notably, N6-isopentenyladenine, indole-3-acetic acid, salicylic acid, cis-zeatin-riboside, trans-zeatin (+)-abscisic acid, cis-zeatin, trans-zeatin-riboside, and N-(3-indolylacetyl)-L-phenylalanine exhibited markedly reduced levels at both the D270 and D300 stages. Conversely, the expression of gibberellin A7 and 12-oxophytodienoic acid showed a significant increase at these stages. Meanwhile, gibberellin A4 displayed an increase at D270 but decreased significantly by D300. Compared to D0, D270 showed downregulation of 5 phytohormones alongside the upregulation of 2 phytohormones; D300 exhibited downregulation of 9 phytohormones, with only 1 phytohormone showing significant upregulation. Compared to D270, D300 displayed the upregulation of 1 phytohormone and the downregulation of 1 phytohormone (Figure 3C). Cluster analysis of DEMs identified two main clusters, with a total of 12 distinct categories of DEMs primarily classified as ABA (1), GA (1), IAA (3), CTK (5), JA(1), and SA (1) (Figure 3D).

**TABLE 1:** Detected metabolites from 9 samples.

Abbreviation	Metabolite	Class
ABA	(+)-abscisic acid	Abscisic acid
GA4	Gibberellin a4	Gibberellin
GA7	Gibberellin a7	Gibberellin
IAA	Indole-3-acetic acid	Auxin
IAA-Ala	N-(3-indolylacetyl)-l-alanine	Auxin
IAA-Asp	Indole-3-acetyl-l-aspartic acid	Auxin
IAA-Leu	N-(3-indolylacetyl)-l-leucine	Auxin
IAA-Phe	N-(3-indolylacetyl)-l-phenylalanine	Auxin
cZ	Cis-zeatin	Cytokinin
cZR	Cis-zeatin-riboside	Cytokinin
DZ	Dihydrozeatin	Cytokinin
IP	N6-isopentenyladenine	Cytokinin
IPA	N6-(Δ2-isopentenyl)adenosine	Cytokinin
tZ	Trans-zeatin	Cytokinin
tZR	Trans-zeatin-riboside	Cytokinin
OPDA	12-oxophytodienoicacid	Jasmonic acid
JA	Jasmonic acid	Jasmonic acid
SA	Salicylic acid	Salicylic acid

**FIGURE 4:**
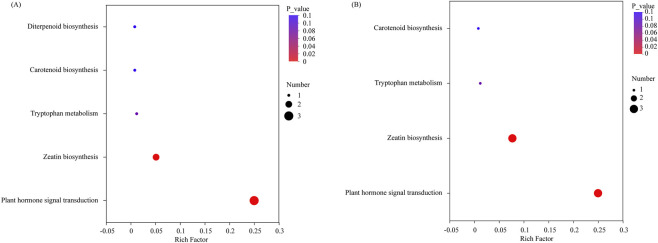
KEGG pathway enrichment analysis of DEMs in different comparison groups. KEGG pathway enrichment analysis of DEMs in D270 vs. D0 comparison **(A)**. KEGG pathway enrichment analysis of DEMs in D300 vs. D0 comparison **(B)**. The enrichment ratio is calculated as num_in_study divided by num_in_pop. The size of the bubbles corresponds to the number of compounds enriched in the respective pathway, while the color gradient indicates the significance level of the enrichment p-values.

To systematically assess the potential roles of DEMs throughout various stratification phases of *S. glabra* seeds in an organized manner, the KEGG annotations of metabolites showing significant variations were categorized based on pathway types defined in the KEGG database. In the D270 vs. D0 comparison, a total of 5 KEGG pathways were identified, while 4 pathways were enriched in the D300 vs. D0 comparison. Notably, the “Plant hormone signal transduction” pathway and the “Zeatin biosynthesis” pathway were significantly enriched in both the D270_vs._D0 and D300_vs._D0 comparisons (Figure 4).

### 3.3 Transcriptome analysis during *Smilax glabra* seed dormancy release

#### 3.3.1 Analysis of transcriptome sequencing results during seed dormancy release

**FIGURE 5:**
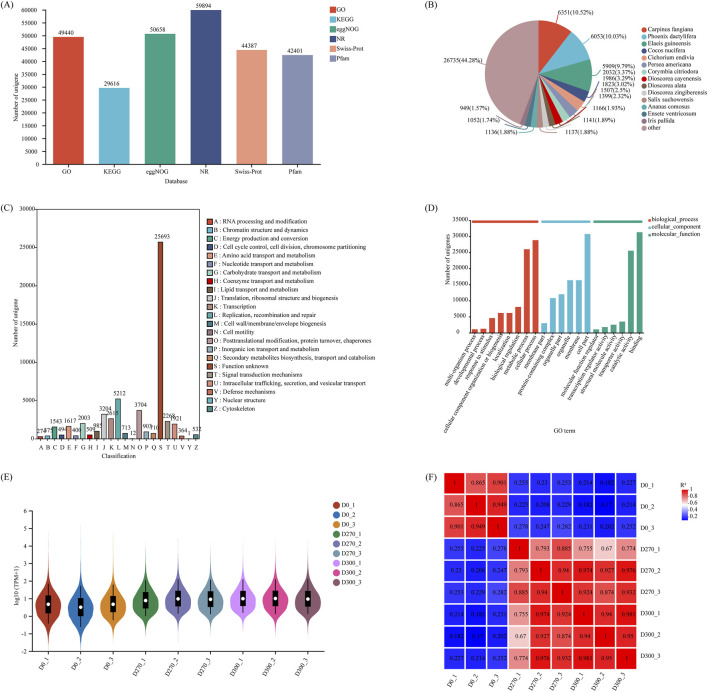
Transcriptome sequencing and quality control of *Smilax glabra* seeds. Functional annotation of total Unigene in various databases **(A)**. Species annotation of genes in the NR database **(B)**. Functional annotation of genes in the EggNOG database **(C)**. Gene Ontology (GO) functional annotation **(D)**. Overall expression level distribution of unigenes across all samples **(E)**. Biological replicate correlation analysis. Different colors represent the magnitude of Pearson correlation coefficients, with higher values indicating stronger correlations **(F)**.

Transcriptome sequencing was carried out on *S. glabra* seeds at various stratification phases. After conducting quality control and data filtering, 384,317,660 clean reads were acquired (Table 2). The Q30 and GC content of the clean reads ranged from 94.54% to 95.09% and 48.24%–51.27%, respectively. The Unigenes obtained from sequencing were comprehensively mapped and annotated against numerous databases, involving GO, KEGG, eggNOG, NR, Swiss-Prot, and Pfam (Figure 5A). Statistical analysis showed that 44,387 Unigenes (32.41%) were annotated in the Swiss-Prot database, while 42,401 Unigenes (30.96%) were annotated in the Pfam database. In the NR database comparison, 59,894 Unigenes (43.73%) were annotated, among which 33,159 Unigenes were categorized as belonging to the top 14 plant species with homologous sequences, accounting for 55.72% of all annotated Unigenes in the NR database. The remaining 44.28% were annotated for other plant species (Figure 5B). Based on comparative analysis against the eggNOG database, 50,658 Unigenes (33.68%) were successfully matched and classified into 23 detailed categories (Figure 5C). For functional annotation, the Unigenes were compared with the GO database. The results showed that 49,440 Unigenes (36.10%) were classified into three primary categories: molecular functions, cellular components, and biological processes, further divided into 53 subcategories (Figure 5D). Finally, 29,616 Unigenes (21.62%) were annotated through comparison and annotation with the KEGG database. The violin-boxplot of gene expression distribution showed relatively stable overall expression levels across different stratification time points (D0270, D300), with highly reproducible results among biological replicates at each time point (Figure 5E). The correlation heatmap demonstrated strong intragroup correlations, while significant differences were detected between D0 and both D270 and D300 samples. Notably, D270 and D300 have a high mutual correlation (Figure 5F).

**TABLE 2:** Summary of generated RNA-seq data.

Sample	Raw reads	Raw bases	Clean reads	Clean bases	Error rate (%)	Q20 (%)	Q30 (%)	GC content (%)
D0_1	42103540	6357634540	41792334	6279994957	0.0127	98.90	94.64	50.35
D0_2	43382656	6550781056	43007682	6457326727	0.0127	98.85	94.54	51.27
D0_3	40561544	6124793144	40274772	6046035427	0.0126	98.91	94.66	50.58
D270_1	43463342	6562964642	43112836	6471119493	0.0124	99.01	95.09	48.51
D270_2	45499024	6870352624	45155128	6778263797	0.0125	98.97	94.86	48.25
D270_3	40380092	6097393892	40033632	6010743050	0.0125	98.99	95.00	48.32
D300_1	48164042	7272770342	47808882	7184239554	0.0124	99.00	95.04	48.24
D300_2	39964502	6034639802	39675368	5962139222	0.0125	99.00	94.99	48.31
D300_3	43796032	6613200832	43457026	6525226299	0.0125	98.99	94.96	48.55

#### 3.3.2 Differential gene expression analysis

**FIGURE 6:**
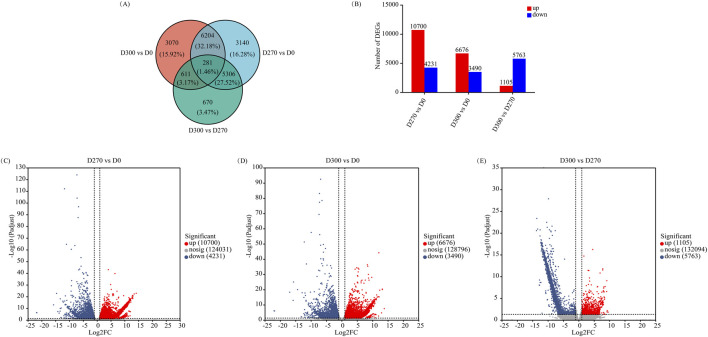
Identification and analysis of DEGs during stratification. Venn diagram of DEGs **(A)**. Number of upregulated and downregulated genes across different comparison groups **(B)**. Volcano plot of DEGs in D270 vs. D0 comparison **(C)**. Volcano plot of DEGs in D300 vs. D0 comparison **(D)**. Volcano plot of DEGs in D300 vs. D270 comparison **(E)**.

A total of 19,282 DEGs were found across the three stages of *S. glabra* seed dormancy release, with 281 genes co-expressed in all stages (Figure 6A). In the pairwise comparisons of D270_vs._D0, D300_vs._D0, and D300_vs._D270, 14,931 DEGs (10,700 upregulated and 4,231 downregulated), 10,166 DEGs (6,676 upregulated and 3,490 downregulated), and 6,868 DEGs (1,105 upregulated and 5,763 downregulated) were identified, respectively (Figure 6B). The volcano plots (Figures 6C–E) visually displayed the overall distribution characteristics of DEGs among the comparison groups. As the stratification time prolonged, the number of upregulated DEGs exhibited a gradual decline, concurrently with a progressive decrease in the total number of DEGs.

#### 3.3.3 Functional characterization of DEGs across different stratification phases

**FIGURE 7:**
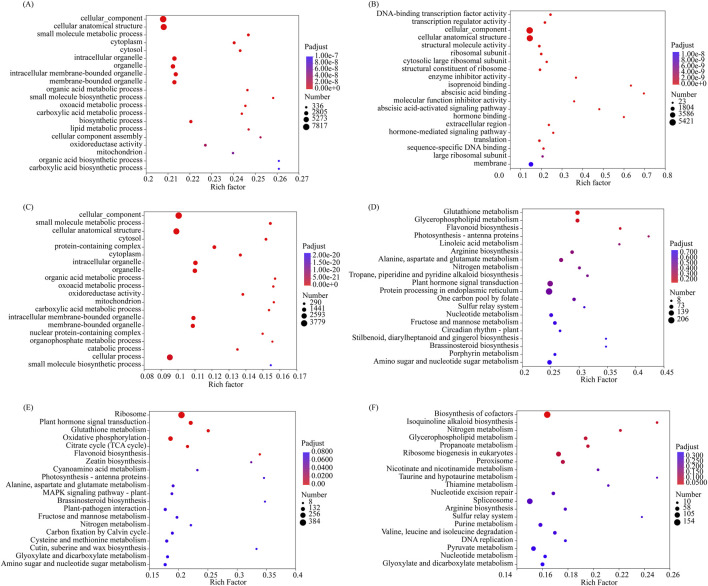
Functional enrichment analysis of DEGs. GO enrichment analysis of DEGs in D270 vs. D0 **(A)**, D300 vs. D0 **(B)**, and D300 vs. D270 **(C)**. KEGG pathway enrichment analysis of DEG in D270 vs. D0 **(D)**, D300 vs. D0 **(E)**, and D300 vs. D270 **(F)**. The Rich factor represents the ratio of DEGs enriched in a specific pathway to the total annotated genes in that pathway. A higher Rich factor indicates greater enrichment. A lower Q-value signifies more significant enrichment.

Gene Ontology (GO) and Kyoto Encyclopedia of Genes and Genomes (KEGG) enrichment analyses were used to determine the biological functions of DEGs at the three stratification phases of *S. glabra* seeds. The observations indicated that in the D270 vs. D0 group, the significantly enriched GO terms for DEGs included carboxylic acid biosynthetic process, organic acid biosynthetic process, and small molecule biosynthetic process (Figure 7A). In the D300 vs. D0 group, the significantly enriched GO terms for DEGs included abscisic acid binding, isoprenoid binding, hormone binding, and abscisic acid-activated signaling pathway (Figure 7B). For the D300 vs. D270 comparison, DEGs were significantly enriched in organic acid metabolic process, oxoacid metabolic process, and mitochondrion (Figure 7C).

KEGG pathway enrichment analysis of DEGs was presented as bubble plots. In the D270 vs. D0 group, the pathways flavonoid biosynthesis, photosynthesis - antenna proteins, and linoleic acid metabolism were highly enriched (Figure 7D). In the D300 vs. D0 group, the pathways ribosome, plant hormone signal transduction, glutathione metabolism, oxidative phosphorylation, and citrate cycle were significantly enriched, while flavonoid biosynthesis and zeatin biosynthesis were highly enriched (Figure 7E). For the D300 vs. D270 comparison, the pathway biosynthesis of cofactors was significantly enriched, and the pathway isoquinoline alkaloid biosynthesis was highly enriched (Figure 7F).

#### 3.3.4 Validation of RNA-seq data reliability using qRT-PCR

**FIGURE 8:**
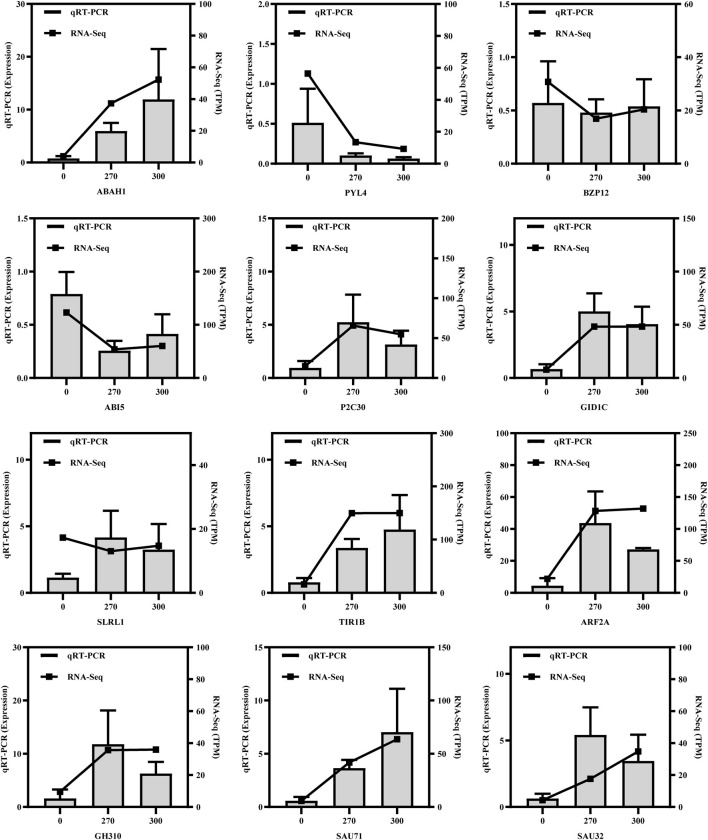
Log2-fold changes of 12 genes in quantitative real-time PCR (qRT-PCR) and RNA-seq; error bars represent the standard deviation of three replicates.

To validate the reliability of RNA-seq data, 12 genes from the plant hormone pathway were chosen for qRT-PCR investigation. As displayed in Figure 8, the majority of detected genes exhibited consistent upregulation or downregulation trends with RNA-seq data, confirming the reliability of RNA-seq data.

### 3.4 Metabolome and transcriptome integrated analysis

#### 3.4.1 The KEGG combined analysis of DEMs and DEGs

**FIGURE 9:**
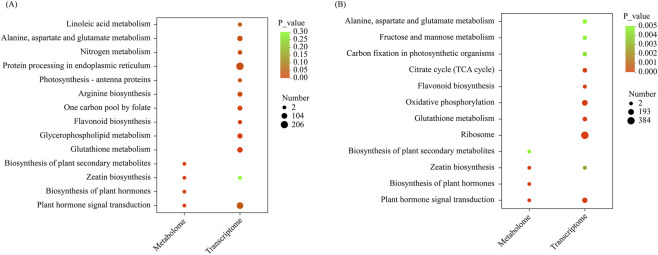
The KEGG combined analysis of DEMs and DEGs: Combined analysis of DEGs and DEMs involved in D270 vs. D0 **(A)**. Combined analysis of DEGs and DEMs involved in D300 vs. D0 **(B)**. The color gradient indicates the significance level of the enrichment p-value. The size of the bubbles represents the number of DEMs or DEGs.

An integrated KEGG enrichment analysis of DEMs and DEGs identified 14 significantly enriched pathways in the D270 vs. D0 group and 12 in the D300 vs. D0 group. Both “zeatin biosynthesis” and “plant hormone signal transduction” were commonly co-enriched pathways in both metabolism and transcription across both groups (Figure 9).

#### 3.4.2 Weighted gene Co-Expression network analysis (WGCNA)

**FIGURE 10:**
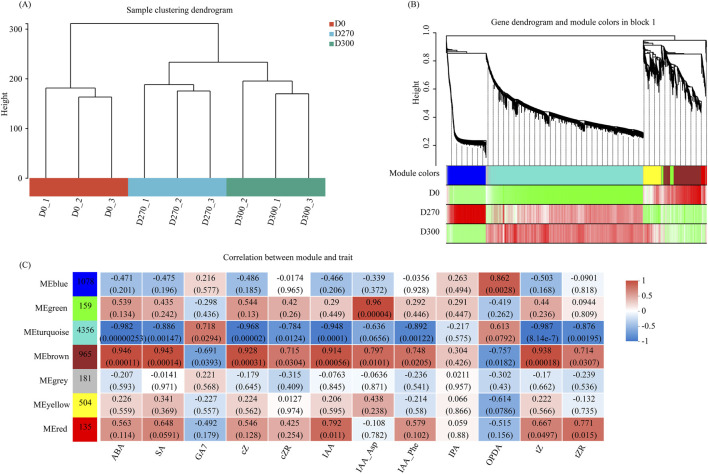
Weighted Gene Co-expression Network Analysis (WGCNA) of genes during stratification stages: Sample clustering dendrogram **(A)**. Hierarchical cluster tree elucidating co-expression modules identified through WGCNA, accompanied by heatmap analysis of sample/module relationships **(B)**. Correlation between modules and metabolites: the color intensity reflects the correlation coefficient between modules and metabolites **(C)**.

To systematically elucidate the dynamic transitions underlying the shift of *S. glabra* seeds from a state of dormancy to germination throughout the stratification process, we employed a weighted gene co-expression network analysis (WGCNA). This analysis examined gene expression patterns across three distinct stratification periods. Genes displaying comparable expression patterns were grouped into separate modules. Furthermore, the study explored potential connections between these co-expression modules and specific biochemical metabolites. Clustering analysis of all samples revealed no significant outliers, indicating that the dataset exhibited strong consistency and robust clustering features (Figure 10A). Modules are typically described as clusters of highly interconnected genes, with genes inside a module having high correlation coefficients in the network. Each branch of the dendrogram represented a module, and each leaf node corresponded to a gene. Modules were distinguished by distinct colors. WGCNA partitioned all differentially expressed genes (DEGs) into seven distinct modules (Figure 10B). We discovered hub genes for each module by mining critical information from them (Table 3). As shown in Figure 10C, during the initial stratification stage (D0), significant positive correlations were found with the red and brown modules, which were closely associated with the high expression of four types (ABA, SA, CTK, and IAA) of plant hormones, including ABA, SA, cis-zeatin (cZ), cis-zeatin nucleotide (cZR), IAA, trans-zeatin (tZ), and trans-zeatin nucleotide (tZR). In the D270 stratification stage, a significant positive correlation was found with the blue module, which was related to the high expression of 12-oxophytodienoicacid (OPDA). During the D300 stratification stage, a significant positive correlation was found with the turquoise module, which was associated with the high expression of two types (GA and JA) of plant hormones: gibberellins a7 (GA7) and OPDA. These findings indicate that the predominant plant hormones correlated with gene expression varied across different stages.

**TABLE 3:** Hub genes of each module.

Module	Hub Genes
Blue	TRINITY_DN3276_c0_g1
Turquoise	TRINITY_DN619_c0_g1
Red	TRINITY_DN0_c17_g1
Yellow	TRINITY_DN2483_c0_g1
brown	TRINITY_DN28_c0_g2
Green	TRINITY_DN18137_c0_g1
Grey	TRINITY_DN5023_c0_g1

### 3.5 Key pathway analysis

#### 3.5.1 Differential expression analysis of genes associated with hormone synthesis, metabolism, and signaling

**FIGURE 11:**
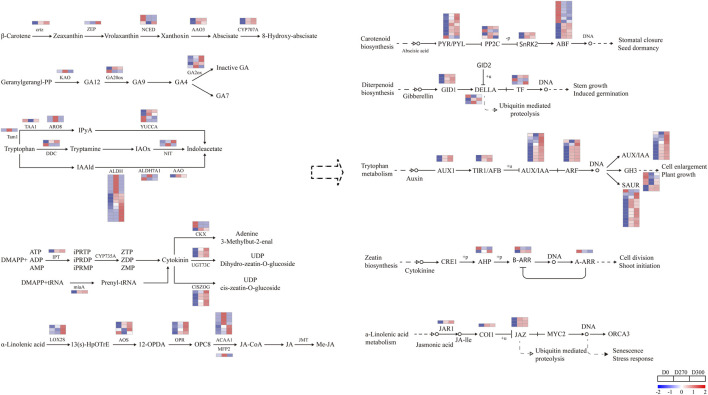
DEGs involved in plant hormone biosynthesis, degradation, and signaling pathways during seed dormancy release in *Smilax glabra*. Gene expression levels are represented by a gradient color scale (log_10_ fold change) across three time points (D0, D270, and D300 of stratification in *Smilax glabra* seeds). The color spectrum ranges from high expression (red) to low expression (blue).

The differential phytohormones identified in this study primarily include ABA, GA, IAA, CTK, and JA. The genes associated with the biosynthesis, metabolism, and signaling pathways of these hormones exhibited differential expression during seed dormancy release, thereby serving as a scaffold for a complex regulatory network that serves as a critical regulator of seed dormancy (Figure 11). In the ABA biosynthesis pathway, genes encoding key enzymes, such as zeaxanthin epoxidase (*ZEP*) and *NCED*, were significantly downregulated at the D270 stage. Conversely, genes encoding the key enzyme in ABA catabolism, ABA 8′-hydroxylase (*CYP707A*), exhibited significant upregulation in both the D270 and D300 stages. Furthermore, genes encoding the negative regulatory factor protein phosphatase 2C (*PP2C*) in the ABA signaling pathway exhibited significant upregulation, resulting in the downregulation of six genes encoding ABA-responsive transcription factors (*ABF*).

Within the GA biosynthesis pathway, one *KAO* gene and one *GA20ox* demonstrated upregulated expression in D270. Additionally, four *GA2ox* genes responsible for degradation also displayed upregulated expression in D270. In contrast, during the D300 stage, two *GA2ox* genes exhibited downregulated expression compared to D270 levels. In the GA signaling pathway, three DEGs encoding gibberellin receptor *GID1* proteins were upregulated, and two DEGs encoding transcription factors (TFs) also exhibited increased expression. Conversely, two DEGs encoding *DELLA* proteins, which serve as negative regulators of GA signaling, were significantly downregulated, thereby promoting gibberellin signal transduction.

In this research, genes associated with the biosynthesis of auxin were predominantly upregulated, particularly those encoding aldehyde dehydrogenase (*ALDH*) enzymes. Additionally, among DEGs associated with IAA signaling transduction, we observed the upregulation of 9 auxin-responsive protein (*AUX*/*IAA*) genes and 7 auxin response factor (*ARF*) genes. Moreover, two of six auxin-responsive *GH3* genes were upregulated. We also identified 12 small auxin-up RNA (*SAUR*) DEGs, including 11 upregulated and 1 downregulated unigene. Notably, 3 DEGs encoding *ABI1/2* were upregulated at D270.

In the CTK synthesis and degradation pathway, only two genes encoding cytokinin oxidase (*CKX*) were downregulated, while the remaining genes (*IPT*, *miaA*, *UGT73C*, *CISZOG*) exhibited upregulated expression. Notably, the number of genes involved in CTK degradation surpassed those responsible for its synthesis. Nevertheless, five DEGs associated with CTK signal transduction and response displayed diverse expression patterns during the release of dormancy in *S. glabra* seeds.

Nearly all JA biosynthesis genes (*LOX2S* and *AOS*) were upregulated, but in the JA signaling pathway, three DEGs encoding the negative regulator jasmonate ZIM-domain (*JAZ*) were upregulated, leading to the inhibition of the JA signaling pathway.

#### 3.5.2 Starch and sucrose metabolic pathways

**FIGURE 12:**
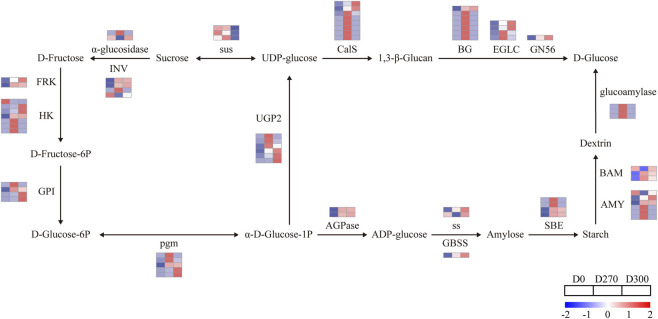
DEGs related to the starch and sucrose metabolism pathway during the release of seed dormancy in *Smilax glabra*. Gene expression levels are represented by a gradient color scale (log_10_ fold change) across three time points (D0, D270, and D300 of stratification in *Smilax glabra* seeds). The color spectrum ranges from high expression (red) to low expression (blue).

This study investigated the regulatory mechanisms of starch and sucrose metabolism, identifying 124 significantly DEGs during seed stratification (Figure 12). Compared to D0, both starch biosynthesis-related genes (including *AGPas, SS, GBSS, SBE*) and starch degradation-related genes (including *AMY, BAM, glucoamylase*) were upregulated at D270 or D300. The sucrose synthase gene sus was downregulated at D300, whereas sucrose degradation genes (*INV*, α-glucosidase) were predominantly upregulated. Additional key DEGs in starch and sucrose metabolism included FP, HK, GPI, pgm, UGP2, CalS, BG, EGLC, GN5, with most showing upregulated expression at D270 or D300, facilitating macromolecule breakdown into glucose. Collectively, these results demonstrate activation of starch and sucrose metabolic pathways during seed dormancy release.

## 4 Discussion

### 4.1 Release of seed dormancy in *Smilax glabra* by stratification

Seed dormancy is defined as the state in which viable seeds fail to germinate under favorable conditions (Bewley et al., 2013). In this study, freshly harvested Smilax glabra seeds exhibited 90% viability, suggesting robust germination capacity. However, fresh seeds failed to germinate (0% germination), thereby confirming the presence of dormancy. After stratification, germination increased significantly, reaching 72.0% at D270 and peaking at 82.7% at D300, which demonstrates that stratification effectively released seed dormancy in this species.

### 4.2 Physiological changes during *Smilax glabra* seed stratification

In studies on seed dormancy and germination in *rice* and *sorghum*, the degradation products of starch and sucrose have been proven to provide sufficient energy for seed germination (Ying et al., 2017; Ju et al., 2025). The key regulatory enzymes involved in starch biosynthesis primarily include UDP-glucose pyrophosphorylase (UGPase), sucrose synthase (SUS), ADP-glucose pyrophosphorylase (AGPase), starch synthase (SS), granule-bound starch synthase (GBSS), and starch branching enzyme (SBE) (Huang et al., 2021; Nougué et al., 2014). This study identified two *AGPases*, 2 *SS*, one *GBSS*, and four *SBEs* that were differentially expressed during the release of dormancy in *S. glabra* seeds. These genes exhibited increased expression at the D270 or D300 stages, suggesting their significant roles in starch biosynthesis in *S. glabra* seeds. While the starch content demonstrated a decreasing trend, suggesting that starch hydrolysis potentially surpasses synthesis. Starch degradation primarily relies on genes encoding α-amylase (*AMY*) and β-amylase (*BAM*) (Zhang et al., 2025). Previous research has indicated that during the release of dormancy in Callery Pear seeds, the expression levels of genes encoding *AMY* and *BAM* exhibit a significant increasing trend (Zhang et al., 2022). In this study, the majority of genes encoding *AMY* and *BAM* in *S. glabra* seeds were upregulated at the D270 and D300 stage, ultimately breaking them down into glucose. The expression patterns of these starch-degrading genes (*AMY*, *BAM*) in Smilax glabra seeds are consistent with the decline in starch content, indicating that stored carbohydrates are mobilized to support metabolic activation and embryo growth during dormancy release in *S. glabra*. Whereas soluble sugar content initially increased and then declined. This pattern may be attributed to the relatively low metabolic activity of the embryo during the early stratification period, during which stored materials like starch are broken down, resulting in the accumulation of soluble sugars. As the stratification duration increases, the radicle begins to penetrate the seed coat, and the accumulated soluble sugars are utilized to support embryo growth and development, leading to a subsequent decline in soluble sugar content. Similar patterns have been observed in *Sassafras tzumu* seeds (Wu et al., 2025). Sucrose degradation is predominantly catalyzed by sucrose invertase (*INV*) and *SUS*. *INV* hydrolyzes sucrose into glucose and fructose, whereas *SUS* degrades sucrose into UDP-glucose (UDPG) and fructose. Under certain circumstances, *SUS* can also catalyze the reverse reaction, synthesizing sucrose from UDP-glucose and fructose, although its degradation rate is faster (Zhai et al., 2021; Zhang et al., 2024; Wang et al., 2025). Additionally, a significant decrease in soluble protein content was found in this study, indicating that proteins are consumed during the dormancy release process of *S. glabra* seeds, consistent with observations in *Macadamia* seeds during stratification (Kang et al., 2025). The degradation of these proteins can provide both nitrogen sources and carbon skeletons for seed germination.

According to metabolic regulation theory, the dormancy release is closely linked to respiratory metabolic pathways, including EMP, TCA, and PPP (Zhong et al., 2023). In this study, as the stratification duration increased, PGI activity significantly decreased, whereas MDH and G6PDH activities showed an overall upward trend. These results demonstrate that the primary respiratory metabolic pathways in *S. glabra* seeds shifted from EMP to TCA and PPP during dormancy release. These findings align with previous studies and offer new proof for the theory proposed by Roberts et al. (Roberts, 1969). Similar trends have been observed in other species, such as increased G-6-PDH activity during stratification in *S. hexandrum* seeds (Ning et al., 2024) and a shift from EMP to PPP/TCA pathways during germination in *Toona sinensis* seeds (Liu et al., 2025).

### 4.3 Phytohormonal regulation during *Smilax glabra* seed stratification

ABA is essential for inducing primary seed dormancy during maturation and preventing the germination of either dormant or nondormant seeds during imbibition in optimum or adverse circumstances, respectively (Krüger et al., 2025). *NCED* is a crucial enzyme in ABA biosynthesis, facilitating the conversion of 9-cis-violaxanthin into xanthoxin. *CYP707A*, as a key gene in ABA degradation metabolism, regulates ABA content by catalyzing the conversion of ABA into 8′-hydroxyabscisic acid (Seiler et al., 2011). In *Arabidopsis thaliana*, four members of the *CYP707A* gene family (*CYP707A1*-*CYP707A4*) have been identified as playing pivotal roles in ABA content regulation (Kushiro et al., 2004). In this study, during the seed dormancy release process, among three DEGs regulating *NCED*, two exhibited consistent downregulation at both 270days and 300days, while one was downregulated at 270days. Additionally, DEGs encoding *CYP707A* were upregulated. These results indicate that ABA biosynthesis decreases while ABA degradation metabolism is activated, with the latter exhibiting a higher activation level, resulting in a decrease in ABA content, consistent with the observed changes in ABA content during hormone measurements. Based on this evidence, it is speculated that *NCED* and *CYP707A* may play important roles in regulating the dormancy release process in *S. glabra* seeds, consistent with the regulatory mechanisms of seed dormancy observed in *A. thaliana* and cereal crops (Tuan et al., 2018; Iwasaki et al., 2022). ABA signal transduction components also affect seed dormancy release (Ali et al., 2022), with the signaling process involving ABA receptors such as the *PYR/PYL/RCAR* family, type 2C protein phosphatase (*PP2C*), and SNF1-related protein kinase 2 (*SnRK2*) (Iwasaki et al., 2022). Modulation of ABA signaling occurs at the transcriptional, translational, and posttranslational levels, where ubiquitination plays a fundamental role in controlling the stability of ABA signaling components by influencing their degradation to adjust and eventually cease this pathway (Moulinier-Anzola et al., 2024). *PP2C* proteins function as negative regulators of the ABA signaling pathway (Xiang et al., 2014; Zhao et al., 2016). To date, several group A *PP2C* proteins in *Arabidopsis*, such as *ABI1*, *ABI2*, *ABI5*, *HAB1*, *HAB2*, *AHG1*, *AHG3*, and *HONSU*, key regulators of seed dormancy, and either overexpressing or losing the function of these genes significantly alter seed dormancy states (Yoshida et al., 2006; Kim et al., 2013; Née et al., 2017; Nishimura et al., 2018; 2025). Similarly, in this study, among four genes regulating *PP2C*, three were significantly downregulated at 270days, resulting in the downregulation of most genes encoding *ABF* transcription factors. This, in turn, promotes dormancy release in *S. glabra* seeds.

GA functions as a key regulator in facilitating seed germination across diverse plant species (Shu et al., 2016; Miura et al., 2023). In this research, one DEG encoding *GA20ox* was upregulated at both D270 and D300, thereby enhancing GA biosynthesis. In this research, a DEG encoding *KAO* and another DEG encoding *GA20ox* were both upregulated at D270, thereby promoting GA4 biosynthesis. Meanwhile, the *GA2ox* gene involved in GA degradation exhibited an upregulation trend, particularly at D300, leading to a transient accumulation of GA4, followed by a subsequent decline. Additionally, no significant differential expression was detected in the key enzyme genes responsible for GA4-to-GA7 conversion. However, hormone metabolism data revealed an increasing trend in GA7 levels, suggesting that its catalytic enzyme activity may be activated through mechanisms such as elevated substrate GA4 concentration and post-translational modifications, potentially involving the activation of post-transcriptional regulatory pathways. GA exerts its effects primarily through the *GID1*-*DELLA*-*SCFSLY1/GID2* signaling cascade (Zhou et al., 2023; Li P. et al., 2024). The GIBBERELLIN INSENSITIVE DWARF1 (*GID1*) receptor facilitates GA signaling by binding to GA, which facilitates the formation of the GA-*GID1*-*DELLA* complex (Gao et al., 2023; Li B. et al., 2024). *DELLA* proteins function as negative regulators within the GA signaling pathway. In the absence of GA, *DELLA* proteins maintain stability and suppress GA responses. Conversely, when GA is present, the GA receptor *GID1* binds to *DELLA* proteins, facilitating the formation of the *GID1*-GA-*DELLA* complex. The complex subsequently interacts with SLEEPY1 (*SLY1*)/*GID2* F-box proteins, leading to the polyubiquitination and subsequent degradation of *DELLA* proteins via the 26S proteasome. This degradation relieves their suppression of GA responses (Saville et al., 2012; Xu and Zhu, 2021). In this study, all *GID1*-encoding genes were significantly upregulated in *S. glabra* seeds, likely playing a positive regulatory role in dormancy release, consistent with findings in *A. thaliana* seeds, where *GID1* accumulation promotes dormancy release (Hauvermale et al., 2015). However, among the three *DELLA* protein-coding genes, two showed an upward expression trend, which differs from findings in *A. thaliana* seeds; the specific molecular mechanism of dormancy release warrants further investigation.

In this research, the key gene *ALDH* involved in the auxin biosynthesis pathway exhibited increased expression, while the levels of free indole-3-acetic acid (IAA) were reduced. This reduction may be attributed to cell activation during the initial germination phase and metabolic remodeling, which resulted in excessive depletion of auxins, causing a pronounced drop in overall auxin levels. Although the expression of auxin biosynthesis-related genes was enhanced during later stages, the synthesis rate proved inadequate to offset the significant early-phase depletion. Consequently, the IAA levels remained below their initial concentrations. Auxin has been found to have dual functions in the regulation of seed germination and dormancy: while low concentrations promote germination, higher concentrations induce dormancy (Shu et al., 2016). In *A. thaliana* seeds, when auxin concentrations are low or auxin signaling is disrupted, auxin-responsive transcription factors *ARF10* and *ARF16* are suppressed by *AXR2/3*, leading to a failure to sustain *ABI3* expression levels, thereby relieving seed dormancy (Liu et al., 2013). During the dormancy release in *S. glabra* seeds, both *ARF* and *IAA* were upregulated, representing a compensatory feedback response to IAA deficiency that enhances signal perception and transduction. These findings differ from observations in *A. thaliana*. N-(3-indolylacetyl)-L-phenylalanine, a conjugated product catalyzed by GH3 (Staswick et al., 2005), exhibited a synchronized decrease with IAA levels. This reduction can be attributed to the dual effects of *GH3* gene downregulation and insufficient IAA substrate availability, which collectively slow the consumption of active IAA.

Other phytohormones, including CTK, BR, JA, ETH, and SA, are also involved in controlling seed dormancy, mainly via modulating the ABA/GA balance (Shu et al., 2016). Isopentenyl transferases catalyze the initial phase of isoprenoid cytokinin synthesis. (Zhao et al., 2024b). After biosynthesis, CTK undergoes glycosylation via glycosyltransferases (such as UGT73C and CISZOG) or is irreversibly degraded by cytokinin oxidase (CKX) (Zhao et al., 2024b). In this study, both *UGT73C* and *CISZOG* exhibited downregulated expression, leading to a decrease in Trans-zeatin, Trans-zeatin-riboside, Cis-zeatin, and Cis-zeatin-riboside levels. Concurrently, N6-isopentenyladenine underwent degradation mediated by *CKX*, and the downregulation of two *CKX* genes attenuated the CTK degradation rate. Despite the overall reduction in CTK content, the upregulation of CTK biosynthetic genes (*IPT* and *miaA*) was observed, driving a shift in CTK metabolism from static storage to dynamic turnover. *A-ARRs* act as negative regulatory components within the CTK signaling pathway. Although the precise mechanism by which *A-ARRs* inhibit CTK signaling remains unclear, it probably entails competing with *B-ARRs* for phosphotransfer and engaging in phospho-dependent interactions with target proteins (Kieber and Schaller, 2018). During the dormancy release process of *S. glabra* seeds, negative feedback regulatory genes (*A-ARR*) were downregulated, whereas the central signaling transduction genes (*AHP*, *B-ARR*) were selectively activated. This activation thereby significantly enhanced the efficiency of cellular responses to low-concentration cytokinin (CTK) signals.

JA has been proposed to inhibit rice seed germination through synergistically acting with ABA and modulating GA signaling pathways at both metabolic and signaling levels (Xu et al., 2025). This study identified 19 DEGs involved in JA biosynthesis. Among these, the upregulated expression of *LOX2* and *AOS* significantly enhanced OPDA biosynthesis, thereby leading to its increased accumulation. However, no downstream DEGs were enriched in the JA-COA pathway. This prevented the efficient conversion of OPDA into JA, ultimately leading to decreased JA levels. Additionally, the upregulated expression of negative regulators *JAZ* in the JA signaling pathway suppressed MYC*2*-mediated activation of transcription in downstream target genes. This indicates that JA negatively regulates the release of seed dormancy in *S. glabra*, which is consistent with previous findings in rice and Callery Pear (Zhang et al., 2022; Xu et al., 2025).

## 5 Conclusion

This study systematically analyzed the physiological dynamics, hormone metabolism characteristics, and molecular regulatory mechanisms involved in the dormancy release process of *S. glabra* seeds. The results demonstrated that alternating temperatures of 25 °C/4 °C effectively broke seed dormancy. During the dormancy release process, seeds consumed soluble proteins and starch, while starch-degrading genes (*AMY* and *BAM*) were significantly upregulated to drive starch conversion into soluble sugars, thereby providing energy for embryo growth. Meanwhile, PGI activity exhibited a marked decrease, while MDH and G6PDH activities showed overall upward trends, reflecting a metabolic shift in the primary respiratory pathways from EMP to TCA and PPP. At the hormone metabolism level, downregulation of *NCED* and upregulation of *CYP707A* synergistically reduced ABA levels, while upregulation of *GA20ox* and *KAO* promoted GA biosynthesis; downregulation of *DELLA* protein-encoding genes relieved GA signal suppression, collectively forming the “ABA decline-GA rise” hormonal balance shift that drives seed germination. Simultaneously, JA biosynthesis genes *LOX2* and *AOS* were upregulated, leading to OPDA accumulation, but OPDA was not converted into JA. Furthermore, the upregulation of the negative regulator *JAZ* blocked JA signal transduction, thereby relieving JA-mediated inhibition of germination. This work significantly enhanced our understanding of the dormancy release mechanisms in *S. glabra* seeds and provided valuable insights for optimizing seed germination control and seedling propagation techniques.

## Data Availability

The original contributions presented in the study are publicly available. This data can be found in the NCBI Sequence Read Archive (SRA) under BioProject accession number PRJNA1357025 and can be accessed at: https://www.ncbi.nlm.nih.gov/bioproject/PRJNA1357025.
