# Supplementary material for: Metabolomic and transcriptomic analyses reveal the mechanisms underlying Smilax glabra seed dormancy release
Source: Front Genet. 2026 May 1;17:1814199. doi: 10.3389/fgene.2026.1814199 (PMC13175537; doi:10.3389/fgene.2026.1814199)
Supplement: Supplementary file 1 [file Table1.docx]

| **Table S1. Primers used for qRT-PCR analysis in this study** | | |  |
| --- | --- | --- | --- |
| Gene | Gene ID | Forward Primer | Reverse Primer |
| *ABAH1* | TRINITY_DN572_c0_g1 | GCTCATTACCAGGACAGGAA | GTTCAGACACATTCACCACAG |
| *PYL4* | TRINITY_DN3508_c0_g1 | GCAGTTTCGTCATTCGTTAC | TTAGCAGCCAATCCAACAT |
| *BZP12* | TRINITY_DN8822_c0_g2 | GCGTCACCAAGGATTCAAG | GCGGCAGAAGAGGATGAT |
| *ABI5* | TRINITY_DN13345_c0_g1 | CAGGGTCCGATTGGTCTT | AGAGCAGAGAAGGGTATTGG |
| *P2C30* | TRINITY_DN545_c0_g2 | CGAAGGGAGATACCACTT | AGAGAGGAAATAAGGACGAA |
| *GID1C* | TRINITY_DN8124_c0_g1 | CCTGGATGGTGACGAAGTA | GATTGATGTCGCTGGGAAC |
| *SLRL1* | TRINITY_DN1260_c0_g1 | TGGTGGTCCTGCGAGTAA | CGTTGCCGATCCATCACA |
| *TIR1B* | TRINITY_DN97_c0_g1 | GGTAACATTACAGGACGACAT | GCTGAACGACTGGAGATG |
| *ARF2A* | TRINITY_DN504_c0_g1 | GTCTATAACATGCCGTCCAA | CTCCACAGCGTTCTCATC |
| *GH310* | TRINITY_DN964_c3_g1 | GATGATGTCGTCGTCGTC | AGGAGAAGGAGAAGGAGAAG |
| *SAU71* | TRINITY_DN3325_c0_g1 | GGCTCCAACAGATGCTAA | GGATGCTGAAGTGGTGAT |
| *SAU32* | TRINITY_DN1315_c0_g1 | CCATGCTGCTGCTTAACAA | CTCCTGTCTGACCCTCTTC |
